# Atomic/molecular layer deposition of europium–organic thin films on nanoplasmonic structures towards FRET-based applications†

**DOI:** 10.1039/d3nr04094a

**Published:** 2023-09-18

**Authors:** Amr Ghazy, Jonas Ylönen, Nagarajan Subramaniyam, Maarit Karppinen

**Affiliations:** a Department of Chemistry and Materials Science, Aalto University FI-00076 Espoo Finland maarit.karppinen@aalto.fi; b Xfold imaging oy FI-00076 Espoo Finland

## Abstract

We present a novel atomic/molecular layer deposition (ALD/MLD) process for europium–organic thin films based on Eu(thd)_3_ and 2-hydroxyquinoline-4-carboxylic acid (HQA) precursors. The process yields with appreciably high growth rate luminescent Eu-HQA thin films in which the organic HQA component acts as a sensitizer for the red Eu^3+^ luminescence, extending the excitation wavelength range up to *ca.* 400 nm. We moreover deposit these films on nanoplasmonic structures to achieve a twentyfold enhanced emission intensity. Finally, we demonstrate the FRET-type energy transfer process for our Eu-HQA coated nanoplasmonic structures in combination with commercial Alexa647 fluorophor, underlining their potential towards novel bioimaging applications.

## Abstract 2

We present a novel atomic/molecular layer deposition (ALD/MLD) process for europium–organic thin films based on Eu(thd)_3_ and 2-hydroxyquinoline-4-carboxylic acid (HQA) precursors.

## Graphical abstract

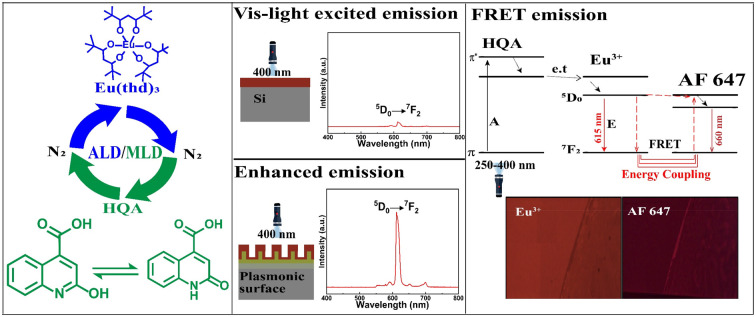

## Introduction

In recent years, the so-called Förster resonance energy transfer (FRET) phenomenon has attracted increasing interest towards various emerging application areas such as biosensing, light harvesting and imaging.^1–6^ In the FRET process, first described by Theodor Förster in 1948,^7^ electronic excitation energy is non-radiatively transferred *via* dipole–dipole interactions from a donor fluorophore to an acceptor fluorophore, which then releases the energy by emitting light at a characteristic wavelength.^8^ This phenomenon has already been employed *e.g.* in biological detection of several viruses, including hantavirus,^9^ Zika virus,^10^ and SARS-CoV-2 (covid-19).^11^

In most of the FRET-based applications, trivalent lanthanide (Ln) ions are used as the donor fluorophores.^12–15^ The Ln^3+^ donors exhibit a long decay time, which allows simple distinction in time for the donor, acceptor and FRET signals.^16–18^ Moreover, the core 4f electrons in Ln^3+^ ions are shielded by the spatially extended 5s and 5p orbitals; this enables the sharp 4f–4f line emission peaks at wavelengths specific to the chosen Ln^3+^ species independent of the coordination environment.^19^ When analysing biological samples, the donor and acceptor fluorophores label the biomolecule of interest, by binding specifically to their target molecule. The FRET signal is then obtained from the molecule which is marked by both fluorophores.^20,21^ The autofluorescence phenomenon typical for biomolecules may somewhat complicate the FRET measurement as it increases the background noise signal, but this can be overcome by using time-resolved FRET measurement mode,^22^ as has been already demonstrated for trivalent lanthanide ions such as Eu^3+^. The Eu^3+^ ions are typically excited at 330–340 nm and their emission maximum is at 615 nm. The emission wavelength is optimal, as it is outside the biological autofluorescence range (400–600 nm) such that the high signal-to-noise ratio is achieved. Moreover, many red emission acceptor fluorophores such as Alexa Flour 647, Dy647, and CyDye are compatible with the Eu^3+^ donors for TR-FRET assays.^23–25^ While the emission wavelength of Eu^3+^ is optimal, the excitation wavelength should be longer, preferably in the visible wavelength range to reduce the phototoxicity and photobleaching effects in biological samples.^26^

The FRET phenomenon occurs when the donor and acceptor moieties are in close proximity (up to 10 nm), and the emission intensity is inversely dependent on the distance between the donor and acceptor.^27,28^ The typically used colloidal Ln^3+^ chelates are not optimal for the process as the donor moieties are suspended in a mobile phase, which is likely to lead to a wide distribution in the donor–acceptor distance. An obvious solution to overcome this issue – but little challenged so far – is to use immobile Ln^3+^ donors fixed *e.g.* into precisely thickness-controlled thin films such that the donor–acceptor distance can be kept in the optimized range.^29^

Here we use for the first time the state-of-the-art gas-phase thin-film technique, atomic/molecular layer deposition (ALD/MLD), to fabricate FRET-active Eu–organic coatings. The unique benefit of the ALD/MLD technique is that it allows atomic-level control of the film thickness and homogeneity even on complex nanostructures.^30,31^ This is important as we will also demonstrate the possibility to enhance the optical fluorescent signal (which is typically weak in bioimaging and diagnostic applications) by utilizing smart nano-plasmonic substrate structures designed to resonate with the Eu^3+^ excitation wavelength. Moreover, we will take a full benefit of the multiple important roles of the organic linker molecules in the films, as they can (i) act as proper spacers between the Eu^3+^ ions to prevent the unwanted concentration quenching effect,^32^ (ii) efficiently sensitize – thanks to their relatively large absorption cross-section – the Eu^3+^ luminescence through a so-called antenna effect,^33^ and (iii) shift the excitation energy range towards the visible wavelengths.

**Fig. 1:**
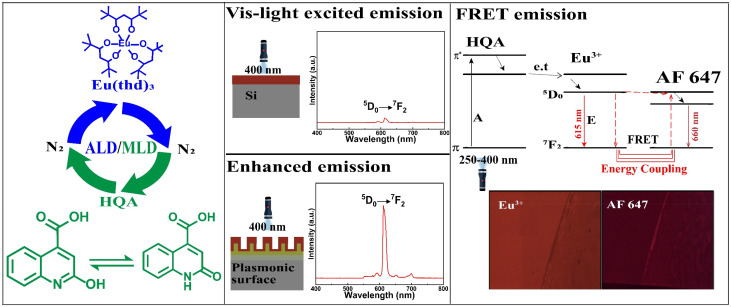
Our research scheme: (left) ALD/MLD allows the fabrication of high-quality Eu-HQA thin films through a cyclic introduction of vaporized Eu(thd)_3_ and HQA precursor pulses separated with N_2_ purge pulses; the two tautomer forms of HQA are shown. (Middle) The HQA linker in the Eu-HQA films acts as a sensitizer for the Eu^3+^ luminescence, such that the films can be excited even with visible light (400 nm). Coating these films on a nanoplasmonic surface enhances the red (615 nm) Eu^3+^ emission by a factor of 20. (Right) Using these luminescent Eu-HQA coated nanoplasmonic structures as the donor fluorophore and Alexa fluor 647 as the acceptor fluorophore, the FRET emission can be realized.

In recent years, several new ALD/MLD processes for luminescent Ln–organic thin films have been developed.^34–39^ In these films the excitation wavelength range has been found to depend on the choice of the organic component, but has nevertheless remained relatively narrow.^40,41^ In this study, we introduce a new organic precursor, 2-hydroxyquinoline-4-carboxylic acid (HQA), which allows us to significantly extend the excitation energy range towards the longer wavelengths. The two tautomer forms of this bi-aromatic HQA precursor are depicted in Fig. 1, which also schematically illustrates our overall research idea.

## Experimental

The Eu-HQA thin films were deposited in a commercial flow-type hot-wall ALD reactor (F-120 by ASM Microchemistry Ltd). The reactor pressure was maintained between 2–4 mbar and in-house produced nitrogen gas was used both as the purging and carrier gas. The two precursors, in-house synthesized Eu(thd)_3_ (thd = 2,2,6,6-tetramethyl-3,5-heptanedione), and commercial 2-hydroxyquinoline-4-carboxylic acid (HQA; Sigma Aldrich, used without additional treatments), were kept in open glass crucibles during the depositions at the following sublimation temperatures: 140 °C for Eu(thd)_3_ and 210 °C for HQA. Initially, when searching for the optimal deposition temperature, the following precursor/purge lengths were applied: 3 s Eu(thd)_3_/4 s N_2_ purge/4 s HQA/6 s N_2_ purge, while the final optimized process carried out at 210 °C constituted of the following precursor/purge sequence: 4 s Eu(thd)_3_/6 s N_2_ purge/4 s HQA/6 s N_2_ purge. Table 1 summarizes the variation ranges for each parameter in our process optimization experiments.

**Table 1:** Deposition parameters used in the ALD/MLD process development

Experiment type	Eu(thd)_3_		HQA		*T*_dep_ (°C)
	Pulse (s)	Purge (s)	Pulse (s)	Purge (s)	
Temperature optimization	3	4	4	6	210–240
Eu(thd)_3_ precursor saturation	1–5	6	4	6	210
HQA precursor saturation	4	6	1–5	6	210

For the initial ALD/MLD process development/optimization the samples were deposited on Si(100) with a substrate size of 20 × 20 mm^2^. Few experiments were also carried out on hand-cut flexible Kapton sheets and quartz glass substrates of 1 mm thickness and a size of 30 × 30 mm^2^ (Finnish special glass). For the FRET characterization, the films were applied as a 25 nm (35 ALD/MLD cycles) coating on nanostructured plasmonic substrates and also for comparison on plain metal substrates at 210 °C, using the optimized precursor pulsing cycle. The Au–metal grating plasmonic substrates were fabricated by electron-beam lithography and evaporation techniques.^42^

The film thickness was determined for each sample using X-ray reflectivity (XRR; X'Pert Pro MPD, PANalytical; Cu Kα), while GIXRD measurements were carried out with the same equipment to show that all our Eu-HQA films were amorphous. Bonding structures were studied by Fourier transform infrared (FTIR) spectroscopy (Bruker Alpha II) performed in transmission mode in the range of 400–4000 cm^−1^. Each sample deposited on silicon was also systematically investigated for the basic luminescence properties; excitation spectra were measured for the 615 nm emission, and the emission spectra with the 250 nm excitation (Edinburgh Instruments FLS1000; continuous-wavelength 450 W Xe lamp (Xe2) as the excitation source; PMT-900 photomultiplier tube as the detector).

For the Eu-HQA coatings deposited on plasmonic nanostructures fluorescence emission spectra were measured using a Leica DM6 microscope, with the output coupled to an Ocean optics HR400 spectrophotometer. The samples were measured in an episcopic illumination setup, through a 20× objective. For the excitation, a 400 nm LED was used, with a 550 nm long-pass filter used for the emitted light. The same microscope setup was used for the fluorescence imaging to analyse the FRET effect. In this case the microscope output was coupled to a scientific camera, and the emission filter was changed to a 615 nm filter with 20 nm FWHM to image the Eu^3+^ emission, and a 660 nm filter with 20 nm FWHM to image the FRET emission.

The FRET phenomenon was demonstrated with a commercially available FRET kit (Alex fluor 647), which can be excited using the 615 nm Eu^3+^ emission, and then the final emission should be seen at 660 nm. For these tests a drop of Alexa647 fluorescent dye was pipetted on the edge of the Eu-HQA coated plain metal and nanostructured plasmonic surface area and allowed to dry in air.

## Results and discussion

### ALD/MLD process development

**Fig. 2:**
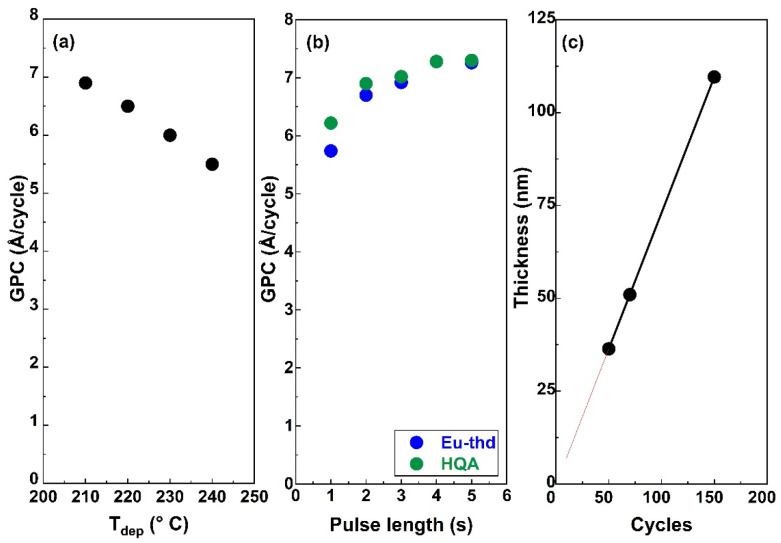
Eu-HQA process optimization: (a) decrease of GPC with increasing deposition temperature; (b) increase and saturation of GPC upon increasing the pulse lengths of the two precursors separately (*T*_dep_ = 210 °C; the other precursor pulse length fixed at 4 s); (c) linear dependence of film thickness on the number of ALD/MLD cycles applied (*T*_dep_ = 210 °C; precursor/purge sequence = 4 s Eu(thd)_3_/6 s N_2_/4 s HQA/6 N_2_).

For the new ALD/MLD Eu-HQA process, we first searched the optimal deposition temperature (*T*_dep_); these experiments were started from 210 °C as the lowest possible deposition temperature, defined by the sublimation temperature of the HQA precursor. The deposition temperature was then increased in the steps of 10 °C, and by 240 °C it became clear that the growth-per-cycle (GPC) value monotonously decreased with increasing *T*_dep_ in a manner typical for most of the ALD/MLD processes, see Fig. 2(a). For the rest of the experiments, we fixed the deposition temperature at 210 °C.

To optimize the deposition process at 210 °C, we investigated the precursor surface saturation behaviours separately for both the precursors by gradually increasing the precursor pulse length (while keeping the pulse of the other precursor unchanged) and monitoring the resultant GPC value. From Fig. 2(b), it can be seen that the film growth rate saturates in both cases when the precursor pulse length is 4 s or longer. Finally, in Fig. 2(c), we demonstrate that the Eu-HQA film thickness can be linearly (*R*^2^ = 1.00) controlled by the number of ALD/MLD cycles applied. Moreover, from the slope of this linear curve, the average GPC value could be determined at 7.29 Å per cycle. Importantly, this GPC value is appreciably high. We attribute this partly due to the relatively large bi-aromatic organic precursor, and also consider it as an indication that the surface reactions between the Eu(thd)_3_ and HQA precursors progress efficiently.

### Bonding structure in Eu-HQA films

**Fig. 3:**
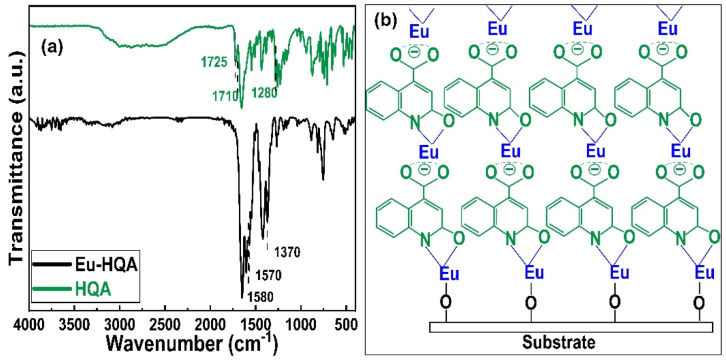
(a) FTIR spectra for HQA precursor powder and a representative Eu-HQA thin film sample. (b) Schematics of the anticipated film growth mode/bonding scheme in our Eu-HQA films.

To confirm the efficient surface reactions, we compare in Fig. 3(a) the FTIR spectra of the organic HQA precursor and our Eu-HQA thin film sample. While the weak peaks between 2950 and 3250 cm^−1^ due to the C–H stretching modes of the organic rings^43^ exist for both HQA and Eu-HQA as expected, it is evident that the dominant peaks of the HQA precursor in the 2200–2900 cm^−1^ range due to the –OH and –NH groups (asymmetric and symmetric stretch modes) are not seen for the Eu-HQA film, indicating that these groups have reacted with the Eu precursor. Also, the peaks due to the C=O bond of the carboxylic acid group seen at 1710 cm^−1^ (asymmetric stretching) and 1280 cm^−1^ (symmetric stretching) in the precursor have been shifted to 1570 and 1370 cm^−1^ in the thin film, thus confirming the coordination between the Eu^3+^ and the carboxylic acid group. To provide further evidence of the completeness of the intended surface reactions we compare the FTIR spectra recorded for the Eu(thd)_3_ precursor and the Eu-HQA thin film (see ESI; Fig. S1†): the thin-film spectrum is free from all spectral features due to the β-diketonate ligand.

The FTIR spectra reveal also further information concerning the bonding structure in our Eu-HQA thin films. Firstly, from the difference between the asymmetric and symmetric stretching modes in Eu-HQA, *i.e. Δ*_*υ*_as_–*υ*_s__ = 1570–1370 = 200 cm^−1^, we may estimate that the bonding between the Eu^3+^ species and the carboxylate group is of ionic type. Tentatively, we believe this ionic bonding could be one of the reasons behind the large GPC value seen for this process. Moreover, since the peaks due to the C2–O bond have shifted from 1725 cm^−1^ (asymmetric stretching) and 1385 cm^−1^ (symmetric stretching) in the precursor spectrum to 1582 and 1420 cm^−1^ for the thin film, we assume that Eu is also coordinated to the oxo group at C2. Additionally, the absence of the NH bending peak seen at 1475 cm^−1^ for the HQA precursor suggests that Eu is also coordinated to the N atom in the pyridine ring. Further confirmation of Eu–O and Eu–N coordination can be concluded from the peaks around 423, 592, and 673 cm^−1^, all of which can only be found in the thin film spectrum (see ESI; Fig. S2 and Table S1†). Fig. 3(b) summarizes these findings and suggestions for the bonding scheme in our Eu-HQA thin films.

### Luminescence properties

**Fig. 4:**
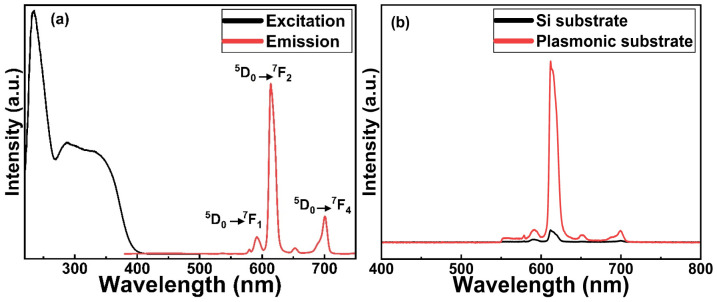
(a) Luminescence properties of Eu-HQA thin films: (a) excitation (*λ*_em_ = 615 nm) and emission (*λ*_ex_ = 250 nm) spectra for Eu-HQA thin film grown on silicon substrate; (b) 20-fold enhanced emission intensity for Eu-HQA thin film grown on a plasmonic substrate in comparison to silicon substrate (*λ*_ex_ = 400 nm).

In Fig. 4(a), we display basic luminescence property characterization data for a 50 nm Eu-HQA thin film grown on silicon. Upon excitation at 250 nm, the Eu-HQA film shows the typical red Eu^3+^ emission, with the main peak located at 615 nm (^5^D_0_ → ^7^F_2_ transition) and the other peaks at 590 nm (^5^D_0_ → ^7^F_1_) and 700 nm (^5^D_0_ → ^7^F_4_). Excitingly, the excitation spectrum (measured for the emission wavelength of 615 nm) is remarkably wide extending through the entire UV range and extending to the visible range from 200 nm to 400 nm. Such a wide excitation range is unprecedented in ALD/MLD-grown lanthanide–organic thin films.^35,40,41,44,45^ For bulk Ln–organic materials similarly wide excitation wavelength ranges have been achieved, but only using remarkably large organic molecules with different substituents and functional groups,^46,47^ which are hardly applicable in gas-phase thin-film depositions as they are difficult to vaporize without decomposition.

Most importantly, the Eu^3+^ emission intensity could be further significantly enhanced by depositing the films on top of a nanostructured plasmonic substrate, the structure of the plasmonic substrate was reported earlier.^48^ As shown in Fig. 4(b), the emission intensity is enhanced by more than 20-fold compared to bare silicon, when measured under the same illumination conditions. The plasmonic substrate is resonating with the excitation light which creates the standing surface waves, with greatly enhanced intensity compared to the excitation light. This allows the maximum absorption of the excitation light in the Eu-HQA thin film leading to the higher emission signal intensity.

### FRET characterization

**Fig. 5:**
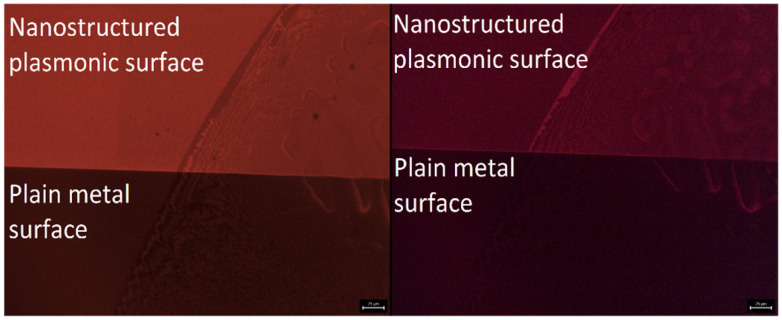
Fluorescence microscope images: (left) 615 nm Eu^3+^ emission, and (right) 660 nm FRET emission, obtained for Eu-HQA + Alexa647 surfaces on nanostructured plasmonic surface (top) and on plain metal surface (bottom). The tilted lines in the images represent the left-side edge of the Alexa647 droplet.

**Fig. 6:**
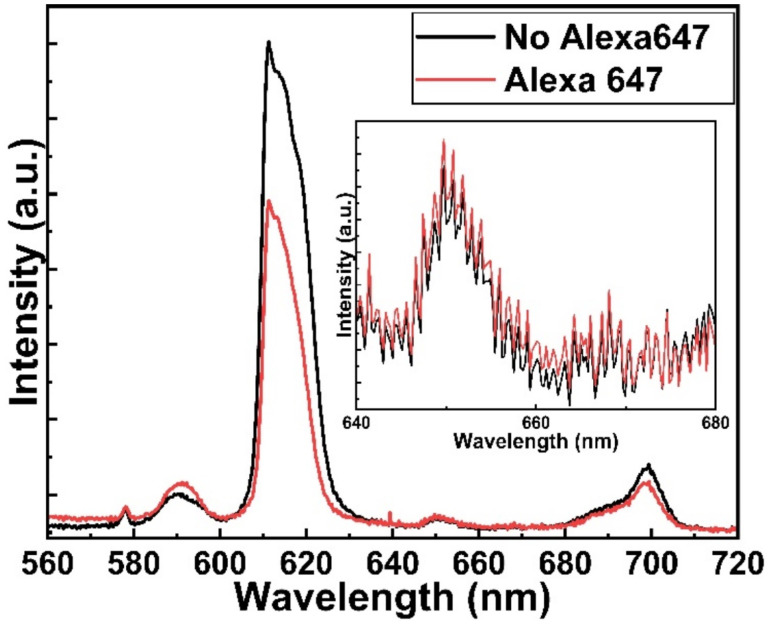
Emission spectra from two different areas on Eu-HQA + Alexa647 surface: pristine Eu-HQA area without Alexa647 and area within which an Alexa647 droplet is located.

Fig. 5 shows the fluorescence microscope images for the emissions obtained from Eu-HQA coated nanostructured plasmonic and metal surfaces (left), and also from the same surfaces after the application of the Alexa647 fluorescent dye drop on top of them. The left-side edge of the Alexa647 droplet is seen as the tilted line in the middle of these images. The Alexa647 droplet is clearly darker in the 615 nm emission image and brighter in the 660 nm emission image, which we believe is clear evidence of the FRET mechanism. It should be also noted that both the Eu^3+^ emission at 615 nm and the FRET emission from AF647 at 660 nm are strikingly stronger in intensity on the nanostructured plasmonic surface compared to the plain metal surface. Finally, we show in Fig. 6 representative emission spectra taken separately from both the pristine area and the Alexa647 droplet area. From this comparison it is seen that the Eu^3+^ emission at 615 nm is clearly lower from areas within which an Alexa647 droplet is located, while the emission around 660 nm is weakly but visibly increased for these areas presumably due to the FRET-like energy transfer.

## Conclusions

We have demonstrated for the first time the FRET phenomenon for Eu–organic thin films grown with the currently strongly emerging ALD/MLD technique. This is remarkable, as it will open the way towards novel bioimaging and biodetection applications.

The success required several important advances regarding the thin-film deposition and substrate development. First of all, a novel ALD/MLD process based on Eu(thd)_3_ and 2-hydroxyquinoline-4-carboxylic acid precursors was developed. This well-controlled process yields high-quality Eu-HQA thin films with a significantly high growth rate of around 7.3 Å per cycle; we attribute the high growth rate to the relatively spacious bi-aromatic organic component, and the specific bonding scheme revealed based on FTIR data involving various bonding modes between the Eu^3+^ ions and the –COOH, –NH and –OH functional groups of the organic precursor. The main highlight of these films is their ability to get excited through the whole UV range starting from 200 nm and extending even to the visible light region up to *ca.* 400 nm.

Independent of the excitation wavelength, the films showed as such the typical red emission expected from Eu^3+^ ions excited in conventional materials typically with 250 nm UV light. Most importantly, the emission intensity could be enhanced twentyfold by coating the Eu-HQA material on specific nano-plasmonic structures that resonate with the Eu^3+^ emission.

Finally, the intended FRET performance was proven by combining the Eu-HQA-coated nano-plasmonic substrates with a commercially available Alex fluor 647, which could be excited with the 615 nm emission and then the final emission was successfully detected at 660 nm. Indeed, this concept now has high potential to be exploited in the bioassay and detection of different viruses.

## Author contributions

The manuscript was written through contributions of all authors.

## Conflicts of interest

There is no conflict of interest to declare.

## Supplementary Material

NR-015-D3NR04094A-s001
