# Supplementary material for: Atomic/molecular layer deposition of europium–organic thin films on nanoplasmonic structures towards FRET-based applications
Source: Nanoscale. 2023 Sep 18;15(38):15865–70. doi: 10.1039/d3nr04094a (PMC10551872; doi:10.1039/d3nr04094a)
Supplement: NR-015-D3NR04094A-s001 [file NR-015-D3NR04094A-s001.pdf]

Electronic Supplementary Material (ESI) for Nanoscale.
This journal is © The Royal Society of Chemistry 2023

Supplementary information

Atomic/molecular layer deposition of europium-organic thin films on nanoplasmonic structures towards FRET-based applications

Amr Ghazy, [a] Jonas Ylönen[b], Nagarajan Subramaniyam[b] and Maarit Karppinen*[a]

[a] Department of Chemistry and Materials Science, Aalto University, FI-00076 Espoo, Finland
[b] Xfold imaging oy, FI-00076 Espoo, Finland
Email: maarit.karppinen@aalto.fi

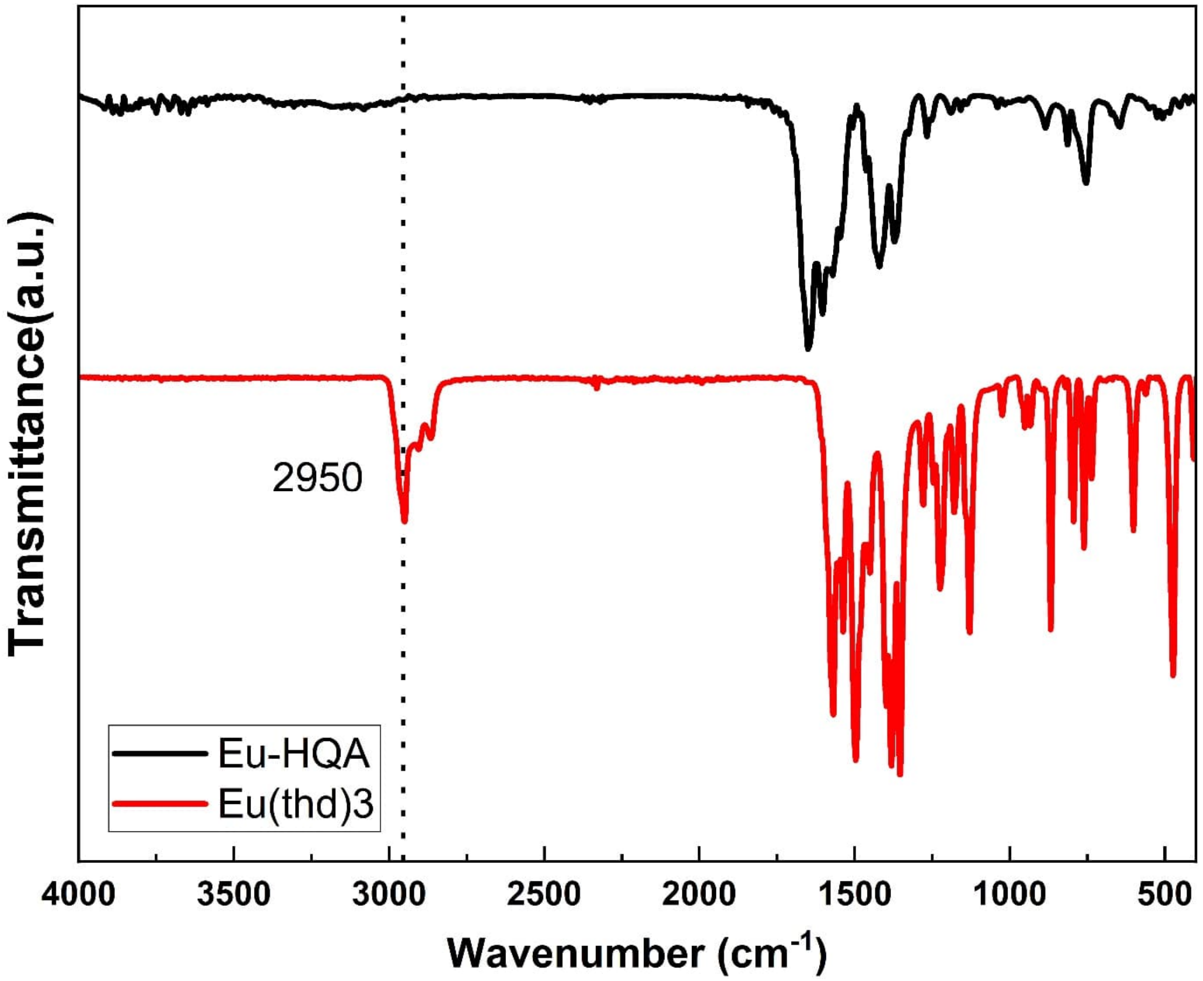

Figure S1 FTIR spectra for Eu(thd) precursor powder and Eu-HQA thin film: comparison of these two spectra clearly shows – upon the Eu-HQA thin film formation – the disappearance of the characteristic β-diketonate ligand peaks in the 2850-2950 cm^{-1} range.

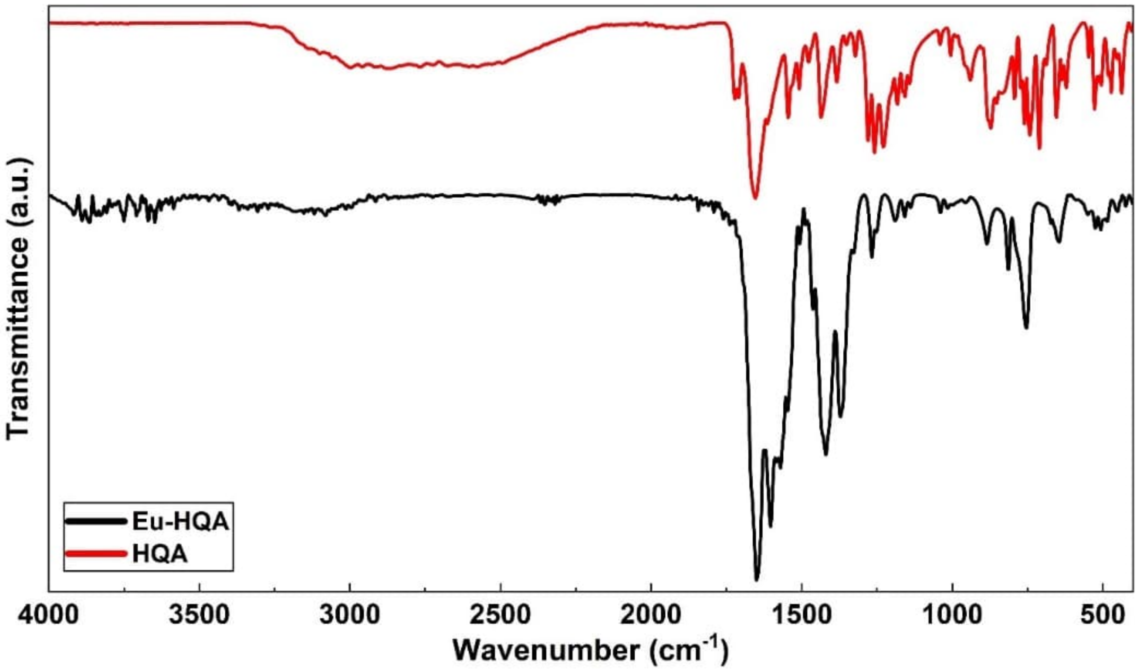

Figure S2 FTIR spectra for HQA precursor powder and Eu-HQA thin film.

Table S1 Detailed interpretations of the FTIR spectral features for HQA and Eu-HQA.

Wavenumber (cm^{-1})	Precursor (HQA)	Thin film Eu-HQA	Interpretation	
			Bond	Mode
423	XXX	423	Eu-O	
~450	453	447	Pyridine ring	In-plane bending
~480	480	480	Pyridine ring	In-plane bending
~505	505	505	Phenyl ring	In-plane bending
592	XXX	592	Eu-O	
651	652	647	pyridine ring	In-plane bending
673	XXX	673	Eu-N	
709	709	XXX	C2=O	Out of plane bending
745	744	752	Phenyl ring	Out of plane bending
761	761	761	Phenyl ring	In-plane bending
880	875	884	C-H in pyridine ring	Out of plane bending
940	940	XXX	NH	Out of plane bending
955	958	955	C-C in pyridine ring	Stretching
987	987	991	C-H in phenyl ring	out of plane bending
1039	1040	1040	C-C in phenyl ring	Stretching
1140	1142	1139	C-H in phenyl ring	In-plane bending
1157	1157	11557	C-H in phenyl ring	In-plane bending
1183	1183	1188	C-H in phenyl ring	In-plane bending
1259	1259	1252	C-C phenyl	Stretching
1278	1278	1270	C-H in phenyl ring	In-plane bending
1321	1321	1326	C-N	Stretching
1349	1349	XXX	C=O	Bending
1371	XXX	1371	C-O-Eu “carboxylate”	Symmetric stretching
1384	1384	XXX	C-OH (C2)	Symmetric stretching
1420	XXX	1420	C-O-Eu (C2)	Symmetric stretching
1434	1434	XXX	C-H in phenyl ring	In-plane bending
1475	1477	XXX	C-N-H	Stretching
1505	1507	1505	C-C in Phenyl ring	Stretching
~1545	1544	1547	C-H in phenyl ring	In-plane bending
1571	XXX	1571	C-O-Eu ”carboxylate”	Asymmetric stretching
1582	XXX	1582	C2-O-Eu	Asymmetric stretching
~1610	1611	1605	C=C in pyridine ring	Stretching
~1650	1650	1647	C=C in phenyl ring	Stretching
1710	1710	XXX	Free carboxylic group C-O	Asymmetric stretching
1723	1723	XXX	C2-OH stretch	Asymmetric stretching
12500-2900	2500-2900	XXX	OH and NH	Stretching (wide peaks)
3000-3250	3000-3250	3000-3250	CH	Asymmetric stretch of both rings (wide peaks)
